# Gamers and Robotic Surgery

**DOI:** 10.21470/1678-9741-2022-0958

**Published:** 2022

**Authors:** Emanuel Ângelo da Silva, Marcelo Luiz Peixoto Sobral

**Affiliations:** 1 Heart Surgery, Faculdade de Medicina, Centro Universitário das Américas, São Paulo, São Paulo, Brazil

Technological literacy that accompanies technology development has become an integral part of our society. In a survey evaluating technology use among school-going individuals from the United States of America and Australia, 96% of students had online access and used technology for various functions, especially video games. Thus, video games have become the most popular multimedia entertainment for them^[1,2]^.

In addition to their entertainment and leisure capabilities, video games may have potential as an education tool, especially in medicine. According to a survey with medical students, 98% of them believed that technology should be better integrated within their medical curriculum, and 80% of them believed that video games can improve their education^[1]^.

**Figure:**
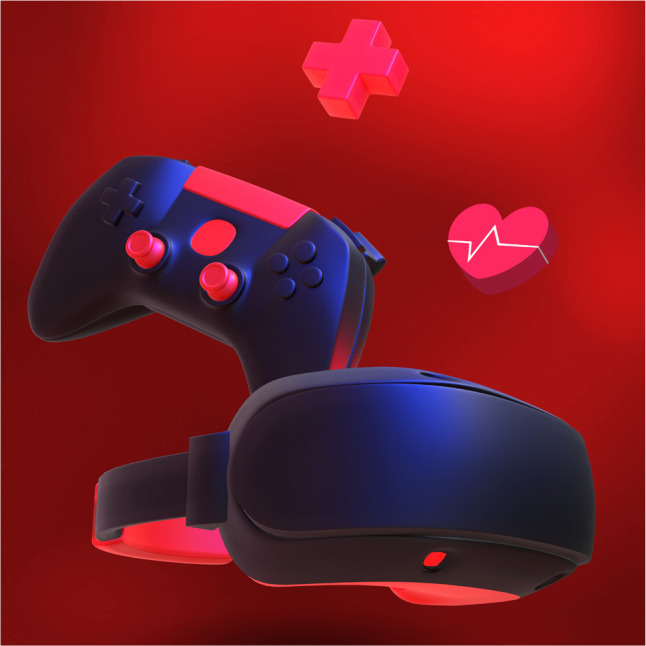

Most interesting of all, video games and surgical procedures share similar skills, such as visuospatial skills and hand-eye coordination; therefore, video games can be a valuable tool for surgical training among medical students^[3,4]^. As is well known, robot-assisted surgery is on the rise, and previous video game experience can be advantageous for budding robotic surgeons^[5]^.

In an observational study, 30 medical students and two interns with a median age of 25 years were recruited and subsequently divided into groups according to their previous gaming experience - gamers (≥ 6 hours of video game/week) *vs.* non-gamers (< 6 hours of video game/week). Participants performed urethrovesical anastomosis by virtual reality simulator on RobotiX Mentor™, which measured performance parameters, and answered a questionnaire for demographics and gaming experience. Players significantly outperformed non-gamers in three of the 24 performance metrics (*P*<0.05), and there was a trend towards better results for seven of the remaining 21 metrics. Previous video game experience > 6 hours/week may give an advantage in simulated robotic surgery^[6]^.

Therefore, training curricula that include video games can help fine-tune the technical interface between surgeons and screen-mediated applications such as robotic surgery. Video games can be a practical teaching tool to help train surgeons^[7]^. Finding high-fidelity ways to teach surgical skills to medical students is vital, as the acquisition of these skills begins at this early point of training^[3,8]^. An important point for further exploration is video game-based training (or VGBT) protocols with hardware and software tailored to surgical skill sets, especially among inexperienced individuals^[5,8,9]^.

However, comprehensive and up-to-date systematic reviews are needed to confirm this. Methodological heterogeneity among included studies limits the ability to make conclusive decisions; thus, future studies with long-term follow-up, larger sample sizes, results stratified by video game characteristics, and up-to-date technology are needed to validate these preliminary results. Also, future studies testing this hypothesis are recommended to develop simulator programs for certification of robotic surgeons.
